# *In Vivo* Confocal Microscopic Evaluation of Corneal Nerve Fibers and Dendritic Cells in Patients With Behçet’s Disease

**DOI:** 10.3389/fneur.2018.00204

**Published:** 2018-03-28

**Authors:** Gulfidan Bitirgen, Emine Tinkir Kayitmazbatir, Gunhal Satirtav, Rayaz A. Malik, Ahmet Ozkagnici

**Affiliations:** ^1^Department of Ophthalmology, Meram Faculty of Medicine, Necmettin Erbakan University, Konya, Turkey; ^2^Department of Ophthalmology, Sorgun State Hospital, Yozgat, Turkey; ^3^Weill Cornell Medicine-Qatar, Education City, Doha, Qatar; ^4^Central Manchester University Teaching Hospitals Foundation Trust and Division of Cardiovascular Sciences, University of Manchester, Manchester, United Kingdom

**Keywords:** Behçet’s disease, corneal confocal microscopy, corneal nerves, corneal sensitivity, dendritic cells

## Abstract

Central and peripheral nervous system involvement may occur during the course of Behçet’s disease (BD). *In vivo* corneal confocal microscopy (CCM) can detect corneal small fiber damage and immune cell density. The aim of this study was to assess central corneal sensitivity, corneal subepithelial nerve plexus morphology and dendritic cell (DC) density in patients with BD. Forty-nine consecutive patients with BD and 30 healthy control subjects were included in this cross-sectional study conducted at a tertiary referral university hospital. Central corneal sensitivity was measured using the contact corneal esthesiometer (Cochet-Bonnet; Luneau, France). The laser scanning CCM (Heidelberg, Germany) was used to quantify corneal nerve fiber density (NFD), nerve branch density (NBD), nerve fiber length (NFL), and DC density. There was a significant reduction in NFD (*P* = 0.001) and NFL (*P* = 0.031) and an increase in DC density (*P* = 0.038) in patients with BD compared to healthy controls, whereas corneal sensitivity (*P* = 0.066) and NBD (*P* = 0.067) did not differ significantly. There was no difference in corneal sensitivity, corneal nerve parameters, or DC density between BD patients with [*n* = 18 (36.7%)] and without a previous history of uveitis (*P* > 0.05 for all). Disease duration [median (IQR), 6.5 (4.0–14.5) years] correlated with corneal sensitivity (ρ = −0.463; *P* = 0.001) and NFD (ρ = −0.304; *P* = 0.034) and corneal sensitivity correlated with NFD (ρ = 0.411; *P* = 0.003) and NFL (ρ = 0.295; *P* = 0.039) in patients with BD. CCM demonstrates corneal sub-basal nerve fiber loss and increased DC density, providing a non-invasive ophthalmic means to identify peripheral neuropathy and inflammation in patients with BD.

## Introduction

Behçet’s disease (BD) is a chronic relapsing vascular-inflammatory disease, typically characterized by oro-genital ulcers, ocular inflammation and cutaneous manifestations. The disease may also involve cardiovascular, pulmonary, articular, gastrointestinal, and neurologic systems (1). Although BD has a worldwide distribution, it is seen more commonly in the Middle East, Far East, and the Mediterranean basin (2).

Neurological involvement in BD ranges from 2.2 to 49% and occurs within the first 10 years of disease (3–6). While brain injury is well documented with parenchymal and vascular involvement (7), there are also studies showing spinal cord and peripheral nerve participation (5, 8–10). An axonal neuropathy has been demonstrated in nerve biopsy and electrophysiological studies in patients with BD (9, 11) and a peripheral neuropathy has also been described in children with BD (12). In a study of 26 patients with BD without neuropathic symptoms, there was electrophysiological evidence of an axonal neuropathy (13). A detailed electrophysiological study of 63 patients with BD showed abnormal nerve conduction and F-wave latencies in 14% (8). In addition, an acute polyradiculoneuritis and mononeuritis multiplex have also been reported in patients with BD (14, 15). Autonomic dysfunction has been described in some (16) but not other (17) studies of patients with BD. A study of 111 patients showed that 19.8% of patients with BD had neuropathic pain, suggestive of underlying small fiber pathology (18).

In relation to eye involvement in BD, most studies have described an optic neuropathy and retinal pathology with maculopathy and retinal neovascularization (19, 20). Studies using optical coherence tomography (OCT) have demonstrated a significant reduction in the thickness of the retinal nerve fiber layer (21), ganglion cell and inner plexiform layers in patients with BD (22), while another study showed an initial increase in the nerve fiber layer in those with a short duration of disease, with thinning in those with a longer duration of disease (23), certainly indicating retinal nerve fiber pathology in patients with BD.

An alteration in corneal biomechanical properties has been demonstrated (24) and central corneal thickness has also been found to be increased in patients with active BD due to inflammation (25). However, there are no studies to date showing corneal nerve involvement in patients with BD. Corneal confocal microscopy (CCM) is a non-invasive ophthalmic imaging technique which allows detailed quantification of the corneal sub-epithelial nerve plexus and dendritic cells (DCs), which are reported to be increased in inflammatory processes (26). We and others have used CCM to demonstrate corneal nerve loss in a broad spectrum of central (27–30) and peripheral (31–34) neurodegenerative conditions and also shown increased DCs in patients with inflammatory neuropathies (35, 36) and multiple sclerosis (29).

To our knowledge, no previous study has used CCM to identify corneal nerve and immune cell alterations in patients with BD. Therefore, the present study has evaluated corneal sensitivity using a contact corneal esthesiometer, and quantified corneal nerve fiber morphology and DC density using CCM in patients with BD.

## Materials and Methods

### Study Subjects

Forty-nine patients (17 males, 32 females) with a diagnosis of BD and 30 healthy control participants (10 males, 20 females) were enrolled in this cross-sectional study undertaken at a tertiary referral university hospital. All patients fulfilled the criteria of the International Study Group for Behçet’s Disease (37). Among patients with BD, 18 (37%) had a previous history of uveitis. Exclusion criteria were active uveitis with topical steroid use, glaucoma, a known history of ocular trauma or surgery, diabetes, other causes of neuropathy, or any other systemic disease that might effect the cornea. The study was approved by the Institutional Review Board and adhered to the tenets of the Declaration of Helsinki. Written informed consent was obtained from all participants after explanation of the nature and possible consequences of the study. All patients underwent a complete ophthalmologic examination. Medical records were reviewed for demographic characteristics, the history of previous episodes of uveitis, duration of the disease, and topical or systemic medications.

### Corneal Sensitivity Assessment

Central corneal sensitivity was measured using a contact corneal esthesiometer (Cochet-Bonnet; Luneau, France). The esthesiometer is based on the principle of pressure transmitted axially by a nylon monofilament, which was applied with a low pressure perpendicular to the center of the cornea. The filament length was progressively reduced from 6 cm in 5-mm steps until the first response occurred. The longest filament length (cm) resulting in a positive response was verified twice and recorded as a measure of central corneal sensitivity (38).

### Corneal Confocal Microscopy

**Figure 1:**
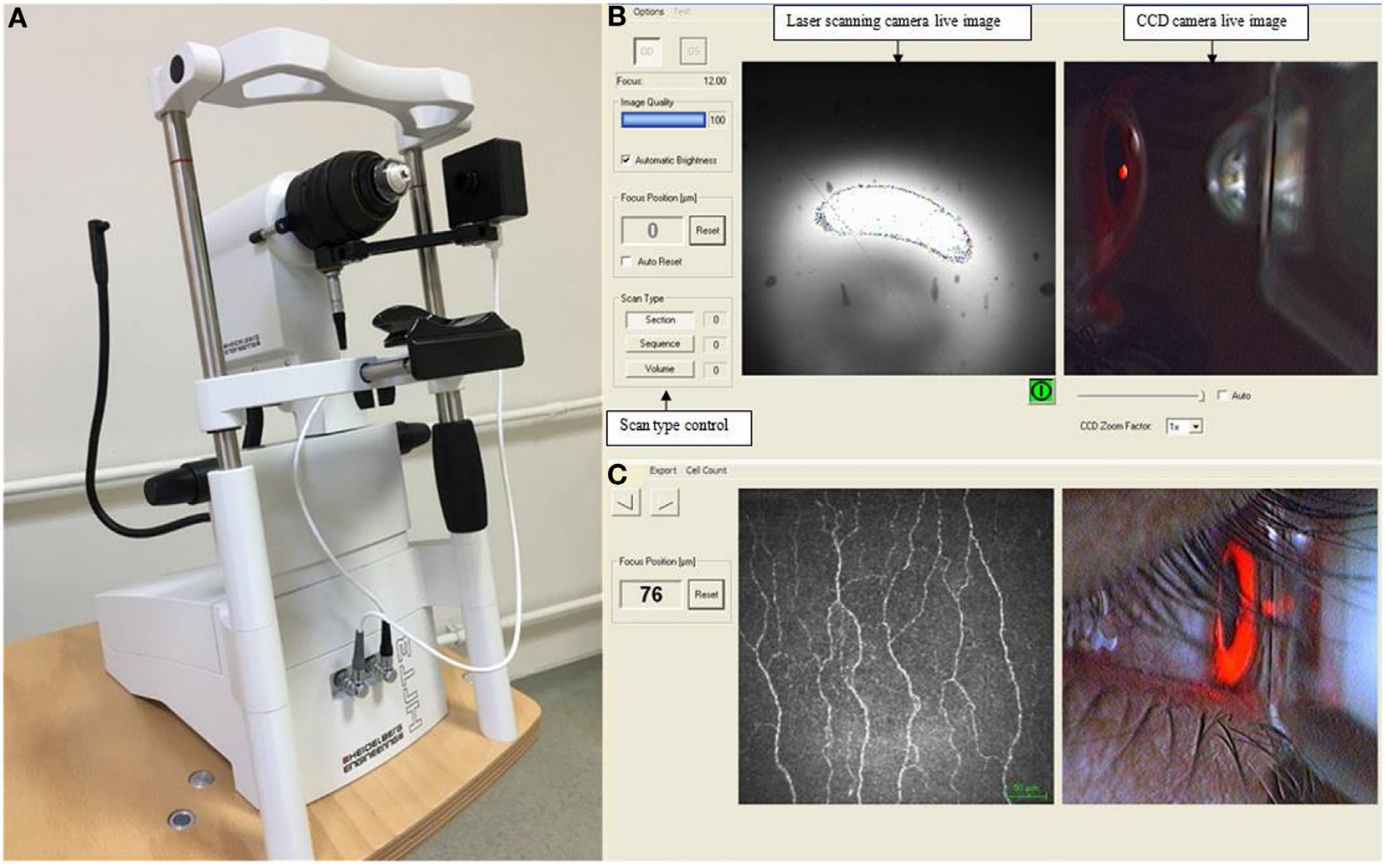
Corneal confocal microscope **(A)**, the viewing screen on monitor during image acquisition **(B)**, and a captured image of the sub-basal nerve plexus using the section mode **(C)**.

Laser scanning CCM was performed in all subjects using the Rostock Corneal Module/Heidelberg Retina Tomograph lll (Heidelberg Engineering, Germany). Three possible examination modes are available in this device (Figure 1). In the “section” mode, a single image is acquired and stored at a time. In the “volume” mode there is automatic acquisition of up to 40 images in consecutive focal planes. The “sequence” mode allows a movie which includes up to 100 images at a selected rate of between 1 and 30 frames per second. In this study, the full thickness of the central cornea was scanned using the section mode and 2D digital images were obtained with a lateral digital resolution of 1 μm/pixel, a depth resolution of 2 μm/pixel and an image size of 400 µm × 400 µm. A previously defined standardized image selection protocol was used (39). Three best-focused nerve plexus images containing the highest, intermediate, and least number of nerve fibers with optimal contrast and without pressure lines or overlapping corneal layers were analyzed from each subject and the average of these results was considered. Automated CCMetrics software, ver. 2.0 (University of Manchester, UK) was used to quantify nerve fiber morphology (40). Three parameters were quantified: corneal nerve fiber density (NFD), the total number of major nerves/mm^2^; nerve branch density (NBD), the number of branches emanating from major nerve trunks/mm^2^; nerve fiber length (NFL), the total length of all nerve fibers and branches (mm/mm^2^) (41). The same images were used to quantify DC density. The number of highly reflective cells with dendriform structures was counted manually and the density was derived as the number of cells/mm^2^ using the proprietary software within the corneal confocal microscope. All the image analyses were performed by a single masked observer. Only one eye of each patient and control participant was included for the analysis as we have previously shown no difference between the right and left eye in healthy control subjects (42) and patients with neuropathy (43). In all except two patients who had undergone cataract surgery in the right eye, right eyes were included.

### Statistical Analysis

Statistical analysis was performed using SPSS ver. 21.0 (Chicago, IL, USA) software. Basic descriptive statistics were calculated on all the data and are reported as the mean ± SD or median (interquartile range), as appropriate. The Pearson χ^2^-test was used to compare categorical variables. Normal distribution of continuous variables was confirmed with the Shapiro–Wilk test. Independent samples *t*-test for normally distributed data and Mann–Whitney *U*-test for non-normally distributed data were used to compare the parameters between the patients with BD and control subjects. The correlations between disease duration, corneal sensitivity, and confocal microscopic parameters were measured using Spearman’s correlation coefficient. For all evaluations, a *P*-value of less than 0.05 was considered statistically significant.

## Results

The mean ages of the patients with BD and control group were 39.9 ± 11.2 years (range 18–64 years) and 41.2 ± 11.5 years (range 18–61 years), respectively. There were no statistically significant differences between the patients with BD and healthy controls for age (*P* = 0.618) and gender (*P* = 0.902). The median value of the time from initial diagnosis of BD was 6.5 years (IQR 4.0–14.5 years).

**Figure 2:**
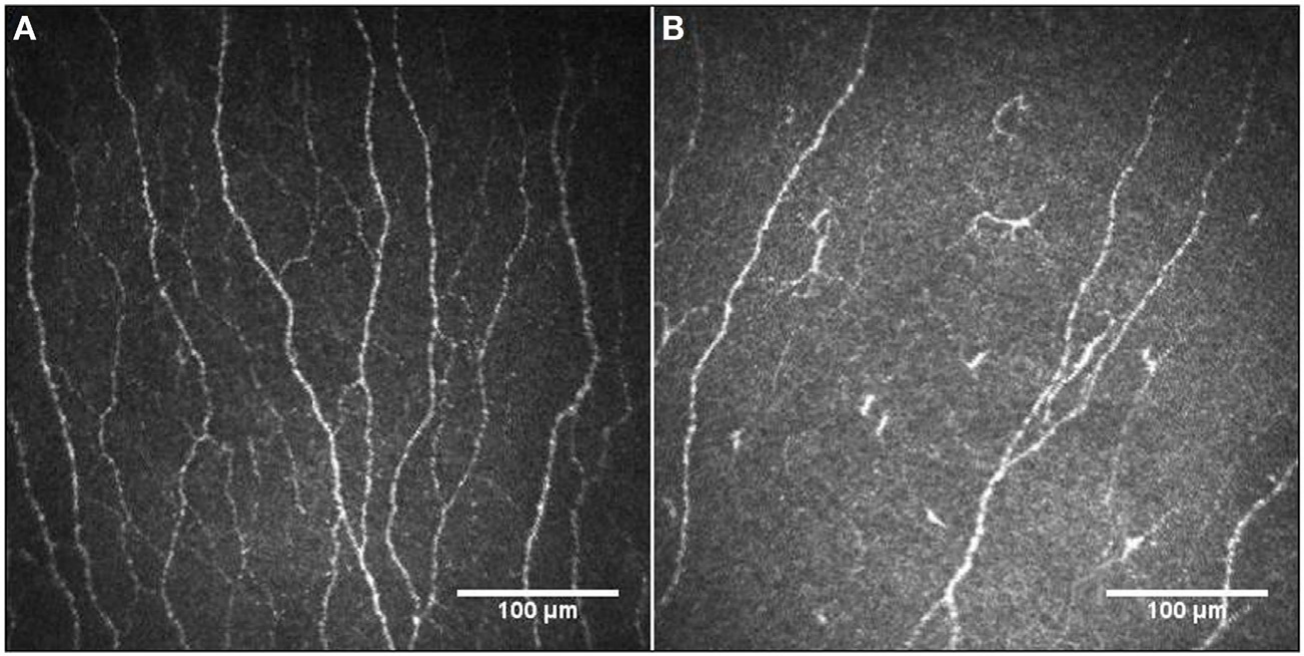
Representative corneal confocal microscopic images of the corneal nerve plexus in a healthy control participant **(A)** and a patient with Behçet’s disease **(B)**, showing reduced nerve fibers and increased dendritic cells.

**Figure 3:**
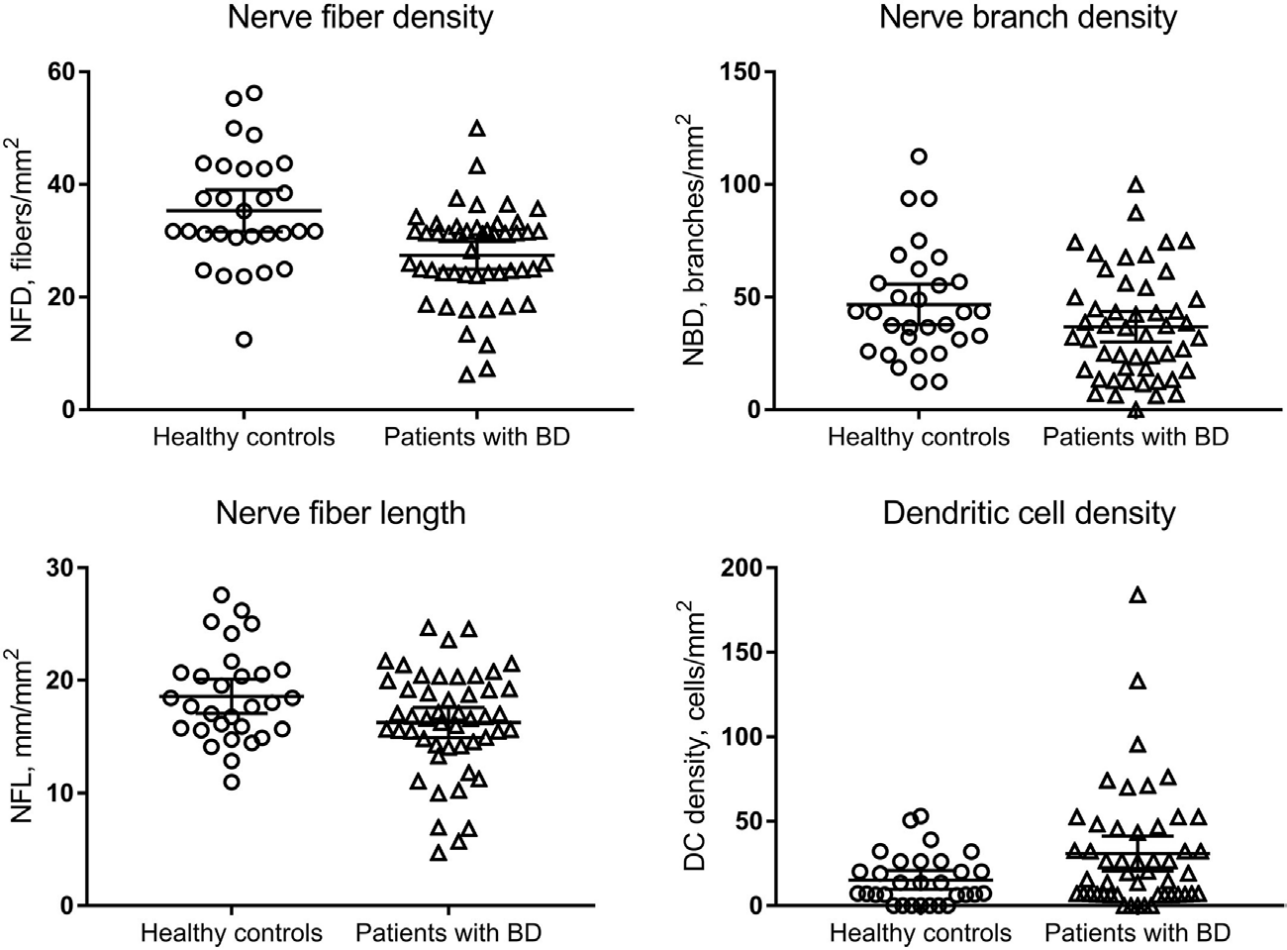
Comparison of corneal subepithelial nerve parameters and dendritic cell (DC) density between healthy control subjects and patients with Behçet’s disease (BD). Patients with BD had a lower nerve fiber density (NFD, *P* = 0.001) and nerve fiber length (NFL, *P* = 0.031), and higher DC density (*P* = 0.038) with no difference in nerve branch density (NBD, *P* = 0.067) compared to controls.

Figure 2 illustrates the CCM images of the central corneal sub-basal nerve plexus in a patient with BD and a healthy control subject. NFD (*P* = 0.001) and NFL (*P* = 0.031) were significantly reduced and DC density was increased (*P* = 0.038) in patients with BD compared to controls (Table 1; Figure 3). There was no significant difference in central corneal sensitivity (*P* = 0.066) and NBD (*P* = 0.067) in BD patients compared to controls. There was no difference in corneal sensitivity, corneal nerve morphology, or DC density between BD patients with and without a previous history of uveitis (data not shown, *P* > 0.05 for all).

**Table 1:** Central corneal sensitivity and corneal confocal microscopic parameters in patients with Behçet’s disease and healthy control group.

	Healthy control group (*n* = 30)	Patients with Behçet’s disease (*n* = 49)	*P*-value
Central corneal sensitivity (cm, mean ± SD)	5.9 ± 0.2	5.6 ± 0.6	0.066^a^
Nerve fiber density (fibers/mm^2^, mean ± SD)	35.6 ± 10.0	27.7 ± 8.6	0.001^a^
Nerve branch density (branches/mm^2^, mean ± SD)	46.8 ± 24.3	36.9 ± 23.9	0.067^a^
Nerve fiber length (mm/mm^2^, mean ± SD)	18.5 ± 4.1	16.3 ± 4.6	0.031^b^
Dendritic cell density [cells/mm^2^, median (IQR)]	10.1 (4.5–26.3)	19.6 (6.3–46.3)	0.038^a^

*^a^Mann–Whitney test*.

*^b^Independent samples t-test*.

Twenty patients (41%) with BD were receiving oral colchicine, 12 (25%) were receiving azathioprine, 4 (8%) were receiving both colchicine and azathioprine, 4 (8%) were receiving both cyclosporine and azathioprine, 2 (4%) were receiving infliximab, and the remaining 7 (14%) were not receiving any treatment. Patients receiving colchicine alone or in combination (*n* = 24) and patients not receiving colchicine (*n* = 25) showed no difference for any of the study parameters (data not shown, *P* > 0.05 for all).

Central corneal sensitivity showed a significant positive correlation with NFD (ρ = 0.411; *P* = 0.003) and NFL (ρ = 0.295; *P* = 0.039) in patients with BD. Disease duration showed a significant inverse correlation with central corneal sensitivity (ρ = −0.463; *P* = 0.001) and NFD (ρ = −0.304; *P* = 0.034).

## Discussion

In this cross-sectional study of 49 patients with Behçet’s disease, a decrease in corneal nerve parameters and an increase in dendritic cell density were observed when compared with healthy controls, with no change in central corneal sensitivity. To our knowledge, this is the first study demonstrating corneal nerve loss in patients with BD and it extends the utility of CCM for identifying peripheral neuropathy in BD.

Neurological involvement is known to be a poor prognostic factor in patients with BD (44). In a large cohort of 530 patients with BD, while central nervous system involvement with parenchymal and vascular pathology affected 10.2%, overt peripheral neuropathy was limited to a single patient (7). However, other studies have reported peripheral neuropathy in 2.1–15.4% of patients with BD (3, 8, 9, 45). Indeed, studies using electrophysiological tests have revealed a subclinical axonal neuropathy in patients with BD (8, 9, 13). Namer et al. (11). have also demonstrated nerve fiber loss in a sural nerve biopsy from a patient with BD and signs of peripheral neuropathy. Neuropathic pain assessed using the Leeds Assessment of Neuropathic Symptoms and Signs index was found in 19.8% of patients with BD, indicative of an underlying small fiber neuropathy (18).

Previous studies have assessed ocular neural tissues in patients with BD using OCT and show a reduction in the retinal nerve fiber layer in patients with Neuro-Behçet’s disease and uveitis (21, 46). More recently, even patients with BD without ocular disease have shown a significant reduction in the retinal nerve fiber layer, ganglion cell layer, and inner plexiform layer, indicative of sub-clinical retinal nerve degeneration (22). Our findings are consistent with previous clinical, histopathological and electrophysiological studies reporting peripheral nerve degeneration in BD (8, 9, 11, 13, 45). We have previously shown that corneal nerve loss is early and comparable to intraepidermal nerve fiber loss (47) and is related to reduced corneal sensitivity and the severity of neuropathy in patients with diabetes (48). In the present study, while we find no overall reduction in corneal sensitivity, it was associated with reduced NFD and NFL as well as disease duration.

Colchicine has been associated with an axonal neuropathy, especially in the presence of renal insufficiency (49). We show no significant difference in corneal sensation or corneal nerve morphology between patients treated with or without colchicine. This is supported by a study of 29 patients with BD which also reported no difference in neurophysiology between patients treated with or without colchicine (50).

Dendritic cells are known to migrate to the central cornea in inflammatory conditions (51). Stettner et al. (36) have reported an increase in DC density in patients with chronic inflammatory demyelinating polyneuropathy and recently we have shown increased DC density around the central corneal nerve plexus of patients with multiple sclerosis (29). We show that DC density is increased in patients with BD, but was not related to disease duration or corneal nerve parameters. Although this study lacks patients with active uveitis, we found no relationship to a history of uveitis.

The limitations of this study are the relatively small sample size and the cross-sectional nature of the study design which precludes conclusions about the natural history of alterations in corneal nerves and DCs in BD. We have also not compared patients with and without Neuro-Behçet’s disease and active uveitis.

In conclusion, we show that CCM can identify subclinical corneal nerve fiber damage and an increase in dendritic cells in patients with BD. Further longitudinal studies are required to determine the diagnostic and prognostic ability of CCM as an imaging biomarker of axonal degeneration and immune activation in patients with BD.

## Ethics Statement

This study was carried out in accordance with the recommendations of the Declaration of Helsinki. All subjects gave written informed consent. The protocol was approved by the Clinical Research Ethics Committee of the Necmettin Erbakan University (2017/844).

## Author’s Note

The preliminary findings of this study were presented at the 51st Annual Congress of the Turkish Ophthalmology Society, October 28, 2017, Antalya, Turkey.

## Author Contributions

All authors contributed sufficiently for being listed as authors of this article. Design of the work: GB, RM, and AO. Data acquisition: GB and EK. Data analysis and interpretation: GB, EK, GS, and RM. Drafting the manuscript: GB and EK. Revising the manuscript: GS, RM, and AO. Final approval: all authors.

## Conflict of Interest Statement

The authors declare that the research was conducted in the absence of any commercial or financial relationships that could be construed as a potential conflict of interest.

## References

[1] Kaklamani VG Vaiopoulos G Kaklamanis PG Behçet’s disease. Semin Arthritis Rheum (1998) 27:197–217. 10.1016/S0049-0172(98)80001-2 9514126

[2] Yazici H Seyahi E Yurdakul S Behçet’s syndrome is not so rare: why do we need to know? Arthritis Rheum (2008) 58:3640–3. 10.1002/art.24146 19035470

[3] Serdaroğlu P Yazici H Ozdemir C Yurdakul S Bahar S Aktin E Neurologic involvement in Behçet’s syndrome. Arch Neurol (1989) 46:265–9. 10.1001/archneur.1989.00520390031011 2919979

[4] Kidd D Steuer A Denman AM Rudge P Neurological complications in Behçet’s syndrome. Brain (1999) 122:2183–94. 10.1093/brain/122.11.2183 10545402

[5] Siva A Kantarci OH Saip S Altintas A Hamuryudan V Islak C Behçet’s disease: diagnostic and prognostic aspects of neurological involvement. J Neurol (2001) 248:95–103. 10.1007/s004150170242 11284141

[6] Talarico R d’Ascanio A Figus M Stagnaro C Ferrari C Elefante E Behçet’s disease: features of neurological involvement in a dedicated centre in Italy. Clin Exp Rheumatol (2012) 30:S69–72. 23009765

[7] Gökçay F Celebisoy N Gökçay A Aksu K Keser G Neurological symptoms and signs in Behçet disease: a Western turkey experience. Neurologist (2011) 17:147–50. 21532383 10.1097/NRL.0b013e3182173379

[8] Akbulut L Gur G Bodur H Alli N Borman P Peripheral neuropathy in Behçet disease: an electroneurophysiological study. Clin Rheumatol (2007) 26:1240–4. 10.1007/s10067-006-0466-0 17149536

[9] Atasoy HT Tunc TO Unal AE Emre U Koca R Esturk E Peripheral nervous system involvement in patients with Behçet disease. Neurologist (2007) 13:225–30. 10.1097/NRL.0b013e31805778d1 17622917

[10] Liu HM Dong C Zhang YZ Tian YY Chen HX Zhang S Clinical and imaging features of spinal cord type of neuro Behçet disease: a case report and systematic review. Medicine (Baltimore) (2017) 96:e7958. 10.1097/MD.0000000000007958 28984755 PMC5737991

[11] Namer IJ Karabudak R Zileli T Ruacan S Küçükali T Kansu E Peripheral nervous system involvement in Behçet’s disease. Case report and review of the literature. Eur Neurol (1987) 26:235–40. 10.1159/000116342 3595663

[12] Metreau-Vastel J Mikaeloff Y Tardieu M Koné-Paut I Tran TA Neurological involvement in paediatric Behçet’s disease. Neuropediatrics (2010) 41:228–34. 10.1055/s-0030-1269909 21210339

[13] Birol A Ulkatan S Koçak M Erkek E Peripheral neuropathy in Behçet’s disease. J Dermatol (2004) 31:455–9. 10.1111/j.1346-8138.2004.tb00531.x 15235183

[14] Bakouche P Guillard A [Polyradiculoneuritis in a developmental flare-up of Behçet’s disease]. Rev Neurol (Paris) (1984) 140:520–2. 6494713

[15] Takeuchi A Kodama M Takatsu M Hashimoto T Miyashita H Mononeruritis multiplex in incomplete Behcet’s disease: a case report and the review of the literature. Clin Rheumatol (1989) 8:375–80. 10.1007/BF02030351 2680237

[16] Kaya EB Yorgun H Akdogan A Ates AH Canpolat U Sunman H Heart-rate recovery index is impaired in Behçet’s disease. Tex Heart Inst J (2009) 36:282–6. 19693299 PMC2720298

[17] Erol T Tekin A Tufan M Altay H Tekin G Bilgi M Autonomic neural control of the cardiovascular system in patients with Behçet’s disease in the absence of neurological involvement. Clin Rheumatol (2012) 31:1499–504. 10.1007/s10067-012-2045-x 22829066

[18] Evcik D Dogan SK Ay S Cuzdan N Guven M Gurler A Does Behcet’s disease associate with neuropathic pain syndrome and impaired well-being? Clin Rheumatol (2013) 32:33–6. 10.1007/s10067-012-2086-1 23001467

[19] Accorinti M Pesci FR Pirraglia MP Abicca I Pivetti-Pezzi P. Ocular Behçet’s disease: changing patterns over time, complications and long-term visual prognosis. Ocul Immunol Inflamm (2017) 25:29–36. 10.3109/09273948.2015.1094095 26727030

[20] Khanfir MS Belfeki N Said F Ben Salem T Ben Ghorbel I Lamloum M Inflammatory optic neuropathy in Behçet’s disease. Reumatismo (2015) 67:156–60. 10.4081/reumatismo.2015.835 27215181

[21] Ucar D Uygunoglu U Dikkaya F Yıldırım Y Yuksel-Elgin C Saip S Retinal nerve fiber layer structure abnormalities in patients with neuro-Behcet’s disease. Graefes Arch Clin Exp Ophthalmol (2015) 253:1181–5. 10.1007/s00417-015-3040-0 25957000

[22] Karadag AS Bilgin B Soylu MB. Comparison of optical coherence tomographic findings between Behcet disease patients with and without ocular involvement and healthy subjects. Arq Bras Oftalmol (2017) 80:69–73. 10.5935/0004-2749.20170018 28591276

[23] Cheng D Wang Y Huang S Wu Q Chen Q Shen M Macular inner retinal layer thickening and outer retinal layer damage correlate with visual acuity during remission in Behcet’s disease. Invest Ophthalmol Vis Sci (2016) 57:5470–8. 10.1167/iovs.16-19568 27760261

[24] Cankaya C Kalayci BN Corneal biomechanical characteristics in patients with Behçet disease. Semin Ophthalmol (2016) 31:439–45. 10.3109/08820538.2014.962168 25392262

[25] Ozdamar Y Berker N Ertugrul G Gurlevik U Karakaya J Ozkan SS. Is there a change of corneal thickness in uveitis with Behçet disease? Cornea (2010) 29:1265–7. 10.1097/ICO.0b013e3181d142b3 20802318

[26] Tavakoli M Boulton AJ Efron N Malik RA. Increased langerhan cell density and corneal nerve damage in diabetic patients: role of immune mechanisms in human diabetic neuropathy. Cont Lens Anterior Eye (2011) 34:7–11. 10.1016/j.clae.2010.08.007 20851037 PMC3017662

[27] Kass-Iliyya L Javed S Gosal D Kobylecki C Marshall A Petropoulos IN Small fiber neuropathy in Parkinson’s disease: a clinical, pathological and corneal confocal microscopy study. Parkinsonism Relat Disord (2015) 21:1454–60. 10.1016/j.parkreldis.2015.10.019 26578039 PMC4671992

[28] Ferrari G Grisan E Scarpa F Fazio R Comola M Quattrini A Corneal confocal microscopy reveals trigeminal small sensory fiber neuropathy in amyotrophic lateral sclerosis. Front Aging Neurosci (2014) 6:278. 10.3389/fnagi.2014.00278 25360111 PMC4199282

[29] Bitirgen G Akpinar Z Malik RA Ozkagnici A. Use of corneal confocal microscopy to detect corneal nerve loss and increased dendritic cells in patients with multiple sclerosis. JAMA Ophthalmol (2017) 135:777–82. 10.1001/jamaophthalmol.2017.1590 28570722 PMC5710203

[30] Petropoulos IN Kamran S Li Y Khan A Ponirakis G Akhtar N Corneal confocal microscopy: an imaging endpoint for axonal degeneration in multiple sclerosis. Invest Ophthalmol Vis Sci (2017) 58:3677–81. 10.1167/iovs.17-22050 28727882

[31] Petropoulos IN Alam U Fadavi H Marshall A Asghar O Dabbah MA Rapid automated diagnosis of diabetic peripheral neuropathy with in vivo corneal confocal microscopy. Invest Ophthalmol Vis Sci (2014) 55:2071–8. 10.1167/iovs.13-13787 24569580 PMC3979234

[32] Bitirgen G Ozkagnici A Malik RA Kerimoglu H. Corneal nerve fibre damage precedes diabetic retinopathy in patients with type 2 diabetes mellitus. Diabet Med (2014) 31:431–8. 10.1111/dme.12324 24117485

[33] Tavakoli M Marshall A Thompson L Kenny M Waldek S Efron N Corneal confocal microscopy: a novel noninvasive means to diagnose neuropathy in patients with Fabry disease. Muscle Nerve (2009) 40:976–84. 10.1002/mus.21383 19902546

[34] Tavakoli M Marshall A Pitceathly R Fadavi H Gow D Roberts ME Corneal confocal microscopy: a novel means to detect nerve fibre damage in idiopathic small fibre neuropathy. Exp Neurol (2010) 223:245–50. 10.1016/j.expneurol.2009.08.033 19748505 PMC2938826

[35] Rajabally YA Stettner M Kieseier BC Hartung HP Malik RA. CIDP and other inflammatory neuropathies in diabetes – diagnosis and management. Nat Rev Neurol (2017) 13:599–611. 10.1038/nrneurol.2017.123 28914883

[36] Stettner M Hinrichs L Guthoff R Bairov S Petropoulos IN Warnke C Corneal confocal microscopy in chronic inflammatory demyelinating polyneuropathy. Ann Clin Transl Neurol (2015) 3:88–100. 10.1002/acn3.275 26900579 PMC4748316

[37] International Study Group for Behçet’s Disease. Evaluation of diagnostic (classification) criteria in Behçet’s disease-towards internationally agreed criteria. Br J Rheumatol (1992) 31:299–308. 10.1093/rheumatology/31.5.299 1581771

[38] Xu KP Yagi Y Tsubota K. Decrease in corneal sensitivity and change in tear function in dry eye. Cornea (1996) 15:235–9. 10.1097/00003226-199605000-00002 8713924

[39] Kalteniece A Ferdousi M Adam S Schofield J Azmi S Petropoulos I Corneal confocal microscopy is a rapid reproducible ophthalmic technique for quantifying corneal nerve abnormalities. PLoS One (2017) 12:e0183040. 10.1371/journal.pone.0183040 28817609 PMC5560560

[40] Dabbah MA Graham J Petropoulos IN Tavakoli M Malik RA. Automatic analysis of diabetic peripheral neuropathy using multi-scale quantitative morphology of nerve fibres in corneal confocal microscopy imaging. Med Image Anal (2011) 15:738–47. 10.1016/j.media.2011.05.016 21719344

[41] Malik RA Kallinikos P Abbott CA vanSchie CH Morgan P Efron N Corneal confocal microscopy: a non-invasive surrogate of nerve fibre damage and repair in diabetic patients. Diabetologia (2003) 46:683–8. 10.1007/s00125-003-1086-8 12739016

[42] Petropoulos IN Manzoor T Morgan P Fadavi H Asghar O Alam U Repeatability of in vivo corneal confocal microscopy to quantify corneal nerve morphology. Cornea (2013) 32:e83–9. 10.1097/ICO.0b013e3182749419 23172119

[43] Petropoulos IN Alam U Fadavi H Asghar O Green P Ponirakis G Corneal nerve loss detected with corneal confocal microscopy is symmetrical and related to the severity of diabetic polyneuropathy. Diabetes Care (2013) 36:3646–51. 10.2337/dc13-0193 23877983 PMC3816900

[44] Akman-Demir G Serdaroglu P Tasci B Clinical patterns of neurological involvement in Behçet’s disease: evaluation of 200 patients. The neuro-Behçet study group. Brain (1999) 122:2171–82. 10.1093/brain/122.11.2171 10545401

[45] Lannuzel A Lamaury I Charpentier D Caparros-Lefebvre D Neurological manifestations of Behçet’s disease in a Caribbean population: clinical and imaging findings. J Neurol (2002) 249:410–8. 10.1007/s004150200031 11967645

[46] Oray M Onal S Bayraktar S Izgi B Tugal-Tutkun I Nonglaucomatous localized retinal nerve fiber layer defects in Behçet uveitis. Am J Ophthalmol (2015) 159:475–81. 10.1016/j.ajo.2014.11.029 25461299

[47] Alam U Jeziorska M Petropoulos IN Asghar O Fadavi H Ponirakis G Diagnostic utility of corneal confocal microscopy and intra-epidermal nerve fibre density in diabetic neuropathy. PLoS One (2017) 12:e0180175. 10.1371/journal.pone.0180175 28719619 PMC5515394

[48] Pritchard N Edwards K Vagenas D Shahidi AM Sampson GP Russell AW Corneal sensitivity as an ophthalmic marker of diabetic neuropathy. Optom Vis Sci (2010) 87:1003–8. 10.1097/OPX.0b013e3181fd6188 21037498

[49] Altiparmak MR Pamuk ON Pamuk GE Hamuryudan V Ataman R Serdengecti K. Colchicine neuromyopathy: a report of six cases. Clin Exp Rheumatol (2002) 20:S13–6. 12371628

[50] Yerdelen D Koc F Uysal H Effects of colchicine on strength–duration properties of sensory and motor axons. Neurol Res (2009) 31:300–3. 10.1179/174313208X346134 18768109

[51] Mayer WJ Mackert MJ Kranebitter N Messmer EM Grüterich M Kampik A Distribution of antigen presenting cells in the human cornea: correlation of in vivo confocal microscopy and immunohistochemistry in different pathologic entities. Curr Eye Res (2012) 37:1012–8. 10.3109/02713683.2012.696172 22667765

